# Next Generation Sequencing Plus (NGS+) with Y-chromosomal Markers for Forensic Pedigree Searches

**DOI:** 10.1038/s41598-017-11955-x

**Published:** 2017-09-12

**Authors:** Xiaoqin Qian, Jiayi Hou, Zheng Wang, Yi Ye, Min Lang, Tianzhen Gao, Jing Liu, Yiping Hou

**Affiliations:** 1 Institute of Forensic Medicine, West China School of Basic Science and Forensic Medicine, Sichuan University, Chengdu, 610041 China; 2 Clinical and Translational Research Institute, University of California, San Diego, La Jolla, CA 92093 USA

## Abstract

There is high demand for forensic pedigree searches with Y-chromosome short tandem repeat (Y-STR) profiling in large-scale crime investigations. However, when two Y-STR haplotypes have a few mismatched loci, it is difficult to determine if they are from the same male lineage because of the high mutation rate of Y-STRs. Here we design a new strategy to handle cases in which none of pedigree samples shares identical Y-STR haplotype. We combine next generation sequencing (NGS), capillary electrophoresis and pyrosequencing under the term ‘NGS+’ for typing Y-STRs and Y-chromosomal single nucleotide polymorphisms (Y-SNPs). The high-resolution Y-SNP haplogroup and Y-STR haplotype can be obtained with NGS+. We further developed a new data-driven decision rule, FSindex, for estimating the likelihood for each retrieved pedigree. Our approach enables positive identification of pedigree from mismatched Y-STR haplotypes. It is envisaged that NGS+ will revolutionize forensic pedigree searches, especially when the person of interest was not recorded in forensic DNA database.

## Introduction

Y chromosome short tandem repeats (Y-STRs) have been used to identify male pedigrees in large-scale crime investigation. The use of Y-STR profiling to narrow down the investigation scale has proven to be successful in solving some cold cases^1^. Y-STRs locating on the Y chromosome non-recombination region (NRY) allow to characterize paternal lineages^2^. Male relatives typically share an identical Y-STR haplotype. This knowledge serves as the basis for the identification of suspect pedigrees by demonstrating haplotype matches^3^. However, due to the high mutation rate of Y-STR, paternal relatives may suffer from inconsistent Y-STR haplotypes. As more Y-STR loci employed, as well as more distant relationship between two profiles is, the probability of encountering any mutation increases^3^ - individuals with discordant Y-STR haplotypes can still be relatives of possible suspects. False exclusion may occur due to potential Y-STR mutations. To solve this issue in forensic pedigree searches, it is of ultimate importance to develop an effective approach that can minimize the false negatives.

According to the Y Chromosome Consortium (YCC) (http://isogg.org/wiki/Genetics_Glossary), the haplotype is defined as a combination of Y-STR alleles inherited directly from male ancestors, and the haplogroup is defined as a set of similar haplotypes derived from the co-ancestors carrying same Y-chromosomal single nucleotide polymorphisms (Y-SNPs). Paternal relatives are also assumed to share identical haplogroups. Since the Y-SNP mutation rate is quite lower (~1.0 × 10^−9^ per year), pedigree signatures are kept much longer at Y-SNPs than Y-STRs^3^. Time estimates of the most recent common ancestor (MRCA) provided by Y-SNP haplogroup analysis are used to study evolution, human origin, and human migrations^4–9^, which leads to predict potential geographic or ancestral origin^10–12^. Searching for relatives or pedigrees with identical ancestral origin is the goal of both pedigree searches and identification of human remains or descendants. The latter has already been implemented using the joint analysis of Y-SNP haplogroup and Y-STR haplotype^13–15^, albeit with rough resolution. Next generation sequencing (NGS) allows us to subdivide the current up-to-date haplogroup tree^16–19^ to trace back to the MRCA within the shortest possible generations. This enables the application of the high-resolution Y-SNP haplogroup analysis to forensic pedigree searches. In crime scene evidence comparisons, the presence of a discordant haplogroup in the reference sample indicates that both samples are derived from different MRCAs and the involved pedigree should be excluded from the scope of further investigations.

In this study, we constructed a high-resolution Y-SNP haplogroup typing panel for forensic pedigree searches. We combined next generation sequencing (NGS), capillary electrophoresis (CE) and pyrosequencing, under the term ‘NGS+’, for typing Y-SNPs and Y-STRs. We further developed a new data-driven decision rule, FSindex, for estimating the likelihood for each retrieved pedigree. The new strategy was validated using 1) 100 father-son pairs; 2) 296 male samples from Han, Tibetan, Uygur and Hui ethnic groups in China and 3) a difficult actual case. Our approach enables positive identification of pedigree from mismatched Y-STR haplotypes. It is envisaged that NGS+ will revolutionize forensic pedigree searches, especially when the person of interest was not recorded in forensic DNA database.

## Results

### Y-STR mutations in 100 father-son pairs

Y-STR haplotyping, that is multiplex analysis of several Y-STRs with capillary electrophoresis (CE), was applied to 100 father-son pairs to test for Y-STR mutations in paternal relatives. One mutation at DYS385 was observed in the minimal haplotype^20, 21^ analysis (including DYS389I, DYS389II, DYS19, DYS391, DYS390, DYS392, DYS385 and DYS393), another mutation at DYS439 was observed through Yfiler haplotype^22^ analysis (including DYS389I, DYS389II, DYS19, DYS391, DYS390, DYS392, DYS385, DYS393, DYS635, DYS458, Y GATA H4, DYS448, DYS456, DYS438, DYS437 and DYS439) in another father-son pair, and a total of 6 mutations occurring in 5 father-son pairs were seen in the 38 loci haplotype analysis (including DYS389I, DYS389II, DYS19, DYS391, DYS390, DYS392, DYS385, DYS393, DYS635, DYS458, Y GATA H4, DYS448, DYS456, DYS438, DYS437, DYS439, DYS533, DYS460, DYS481, DYS443, DYS444, DYS446, DYS459, DYS510, DYS520, DYS522, DYS527, DYS531, DYS549, DYS552, DYS557, DYS587, DYS622, DYS630 and Y GATA A10) (details in Table 1, Supplementary Table S1). When rapidly mutating (RM) Y-STRs were introduced to the analysis, Yfiler Plus haplotype^23^ analysis (including DYS389I, DYS389II, DYS19, DYS391, DYS390, DYS392, DYS385, DYS393, DYS635, DYS458, YGATAH4, DYS448, DYS456, DYS438, DYS437, DYS439, DYS460, DYS481, DYS533, DYS576, DYS627, DYS518, DYS570, DYS449 and DYF387S1) showed that 5 of the 8 mutation events were observed at RM Y-STRs, accounting for 62.5% of all mutations (Table 1, Supplementary Table S1).

**Table 1:** Ten father-son pairs with eleven mutations were tested with NGS+.

	Haplotype Name	Sample	Locus	Allele(Father → Son)	Haplogroup
	Minimal	Han86	DYS385	13,20 → 13,19	O2a1c1a1a1
	Yfiler	Han27	DYS439	12 → 13	O2a2b1a1a6
Yfiler Plus	conventional Y-STR	Han13	DYS481	26 → 27	O2a2
	RM Y-STR	Han57	DYS449	32 → 33	O1a1a1a1a1a1
	RM Y-STR	Han64	DYS627	23 → 22	O2a1c1a1a1a1
	RM Y-STR	Han73	DYS449	32 → 33	O2a1c1a1a1
	RM Y-STR	Han85	DYS570	20 → 19	Q
	RM Y-STR	Han96	DYS518	40 → 41	C2c1
	38 Y-STRs	Han41	DYS549	13 → 12	O2a2b1a1a1
		Han86	DYS552	25 → 26	O2a1c1a1a1
		Han97	DYS444	12 → 13	O2a2b1a1a5

### Y-SNP haplogroup typing with NGS+

Due to its extremely low mutation rate, Y-SNP is an optimal marker for forensic pedigree searches. We screened Y-SNPs from the Y Chromosome Haplotype Reference Database (YHRD) (https://yhrd.org), International Society of Genetic Genealogy (ISOGG) (http://www.isogg.org/tree/index.html) and the 1000 Genomes Project (https://www.ncbi.nlm.nih.gov/variation/tools/1000genomes/) and constructed a high-resolution haplogroup tree aiming at major Chinese populations for forensic pedigree searches.

A 74 Y-SNPs panel was designed according to our haplogroup tree for the Chinese population (Thermo Fisher White Glove Approach ID: IAD97812). 296 unrelated individuals randomly sampled from Han, Tibetan, Uygur and Hui ethnic groups in China were genotyped with the Ion Torrent PGM^TM^ System (Life Technologies, USA). A total of 33 terminal haplogroups were observed (Supplementary Figures S1–S2). These Y-SNPs covered all major haplogroups for the Chinese population and provided the basic structure of the haplogroup tree for forensic use. However, the haplogroup tree was insufficient to reduce the size of potential pedigrees and further subdivision was needed. By pyrosequencing, a total of 67 Y-SNPs supposed to be polymorphic in the Chinese population were selected. 53 of them were observed with two different alleles among 296 individuals, thus showing polymorphisms in the Chinese population. As the total number of Y-SNPs increased to 139 (two were excluded according to the latest ISOGG Y-SNP haplogroup tree version 2017), 73 haplotypes were observed among 296 individuals (Supplementary Figures S3–S4). Most of haplogroup frequencies among Chinese population were less than 0.05.

### High-resolution joint analysis of NGS+

In this study, the Y-STR haplotype analysis was based on CE, while the high-resolution Y-SNP haplogroup analysis was based on NGS and pyrosequencing. Y-SNP haplogroup analysis showed that all of the 100 father-son pairs had identical Y-SNP genotypes, including those with Y-STR mutations (Table 1). Among the 296 unrelated individuals, two samples from Han and Tibetan, respectively had an identical Yfiler haplotype but discordant haplogroups. The Han sample carried haplogroup O2a2b1a1a and the Tibetan sample carried haplogroup O2a2b1a1a1 (Supplementary Figure S4). Most of the unrelated individuals with the identical haplogroup were further differentiated by the Yfiler haplotype. Only 7 groups of unrelated individuals had coincidentally identical haplogroups and haplotypes, and each was from the same ethnicity (Supplementary Figure S4).

### Biostatistical analysis with FSindex

Biostatistical analysis was conducted to estimate the likelihood of the suspect pedigree came from the retrieved pedigrees. A likelihood formula, FSindex, was proposed based on our forensic pedigree search approach. Haas *et al*.^15^ presented two different methods for Y-STR likelihood calculations, which were modified and applied to the analysis of Y-STR and Y-SNP respectively in this study (see Supplementary Note). The new hypotheses are proposed as follows: the null hypothesis is that the suspect does not come from the involved reference pedigree but fortuitously shares the same Y-SNP haplogroup and similar Y-STR haplotype as well as the involved pedigree can be excluded from the scope of further investigation; the alternative hypothesis is that the suspect maybe comes from the involved reference pedigree and Y-STR mismatches are caused by mutations as well as the involved pedigree has to be included in the scope of further investigation. The FSindex likelihood formula for forensic pedigree searches is as follows:

$${\rm{F}}{\rm{S}}{\rm{i}}{\rm{n}}{\rm{d}}{\rm{e}}{\rm{x}}=\frac{L({H}_{A}:D)}{L({H}_{0}:D)}=\frac{(\begin{array}{c}t\\ m\end{array})\cdot {(1-X)}^{(t-m)}\cdot {X}^{m}\cdot {S}^{n}}{A\cdot B+{P}_{AB}\sqrt{A(1-A)\cdot B(1-B)}}$$

where t is the total number of Y-STR loci used, m is the number of mismatched Y-STR loci, and each mismatch is calculated as one repeat unit difference. X equals the average Y-STR mutation rate times the maximum meiosis of the reference pedigree with the mutation rate set as 0.0028 per locus per generation^12^. n is the number of Y-SNPs used, S equals one minus the per mutation rate of Y-SNP of 0.000000001^12^, reflecting the necessary lack of mutation at all the detected Y-SNPs. A denotes the Y-STR haplotype frequency of the reference pedigree in the target population. B denotes the Y-SNP haplogroup frequency of interest in the target population. P_AB_ is the correlation coefficient and set as 0.9969, according to the four studied ethnic groups (Supplementary Figure S5). As a recommendation, all pedigrees with an FSindex greater than 1 should be included in the scope of further investigations, otherwise pedigrees should be ruled out. Preconditions of the use of the FSindex detailed in Supplementary Note.

### Forensic pedigree searches with NGS+

Our forensic pedigree search approach was applied to assist in solving an actual case. There was no Yfiler haplotype match between the crime scene evidence and the local 97 reference pedigrees (194 samples). The police investigators faced with the dilemma of whether there was a necessity to keep searching the suspect locally.

**Figure 1:**
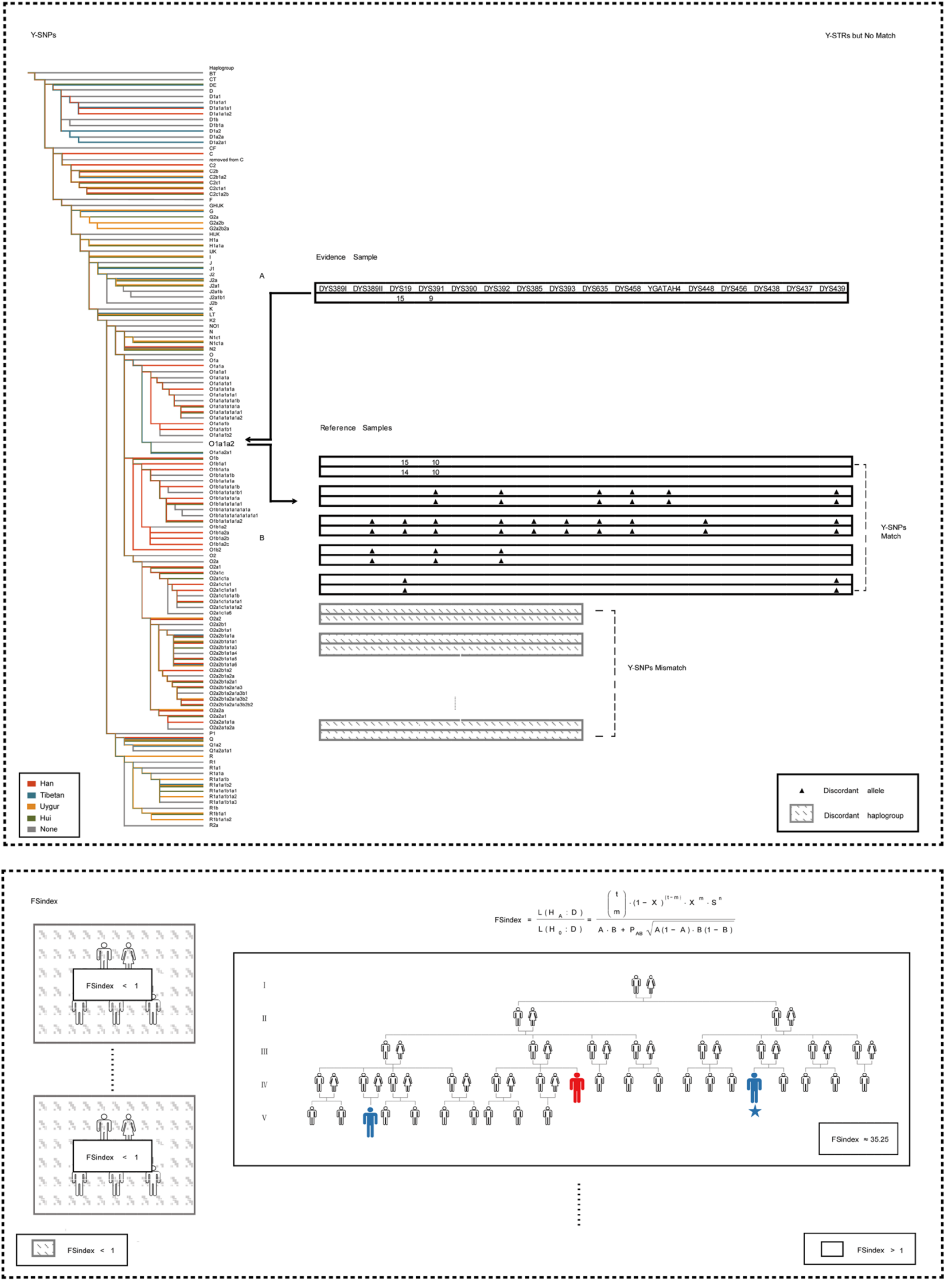
Case application of forensic pedigree search strategy with NGS+. In the first box, all the reference pedigrees mismatched to the Y-STR haplotype of crime evidence were analyzed further by defining the Y-SNP haplogroup and determining whose haplogroup was identical to that of the crime scene evidence. (**A**) Y-SNP haplogroup analysis of the crime evidence. (**B**) Y-SNP haplogroup analysis on all the reference pedigrees. The second box shows the biostatistical analysis based on FSindex. All the male members of the pedigree with FSindex >1 were examined in the further investigation. Reference individuals are represented in blue and the profile used for FSindex computation is marked by a pentagram. The source of the crime evidence is represented in red.

NGS+ results showed that Y-SNP genotypes of five pedigrees were identical with the crime scene evidence, carrying haplogroup O1a1a2-CTS52. We calculated the FSindex of these five pedigrees, respectively. As very few haplogroup frequencies exceeded 0.05 in the overall Chinese population in the high-resolution Y-SNP haplogroup analysis, the frequency of the haplogroup O1a1a2-CTS52 was conservatively estimated as 0.05. All male members of the pedigrees with an FSindex greater than 1 were recommended to undergo autosomal STR analysis. This was successful in revealing a matching suspect. The suspect pedigree had two branches of closely related descendants (Fig. 1), and A7 and B7 were sampled as representatives. Discrepancies were observed at DYS391 and DYS19 in Yfiler haplotype analysis (DYS391: the crime scene evidence was 9 while A7, B7 were 10; DYS19: the crime scene evidence was 15 while A7 was 15 but B7 was 14). However, the Y-SNP haplogroup analysis results were identical. In this case, the maximum number of meiosis was taken as 7, herein the FSindex was calculated as 35.25 (Table 2).

**Table 2:** Representatives of the potential pedigree and the sample from the crime scene were tested using our forensic pedigree search approach with NGS+.

Sample		DYS391	DYS19	Haplogroup	FSindex
Sample from the crime scene		9	15	O1a1a2-CTS52	35.25
Sample from the reference pedigree	A7	10	15	O1a1a2-CTS52	
	B7	10	14	O1a1a2-CTS52	

Discordant loci and alleles are shown.

## Discussion

Y-STR has been successfully used to differentiate male DNA in sexual assault cases in forensics^24, 25^. Although Y-STR haplotypes are hypervariable and hypermutable^26^, they are pedigree-specific and therefore can only be used for pedigree inference, rather than personal identification. Y-STR haplotype analysis of 100 father-son pairs indicated that Y-STR mutations were unavoidable even in just one generation. The more loci were used, the more mutations were observed. In this study, 5 mutation events were observed among 7 RM Y-STRs. RM Y-STRs typing leads to an increasing number of discrepancies observed between close relatives^27, 28^ and may cause a dilemma to determine whether non-kinship or kinship with mutations. Generally, a Yfiler haplotype (17 Y-STRs) can provide sufficient information^29, 30^ without showing too many discrepancies between relatives. Mutations also occur as the number of meiosis events increase or the genetic relationship becomes more distant^31^. Undoubtedly, the phenomenon will be more common in pedigree searches since it is more likely that the suspect and the reference sample are distantly related. When more possible mismatches between two profiles from relatives are expected, the investigation will be placed on a larger scale. China has the largest population in the world and each pedigree has been through many generations. Thus, large-scale investigations are time- and effort-consuming and less successful than expected. It is important to find a more efficient approach of searching potential pedigrees. Liu *et al*.^31^, after testing more than 20,000 individuals, proposed the hypotheses that two Yfiler profiles with less than two mismatched loci were potentially related, and two Yfiler Plus profiles with no more than four mismatched loci or five mismatched steps were potentially related. These theories served as guidelines for forensic pedigree searches, but still required a more solid scientific foundation to support. In this study, we proposed a new forensic pedigree search strategy with NGS+. The mutation rate of Y-SNP is 100000 times lower than Y-STR^32^, therefore, we focused on constructing a high-resolution Y-SNP haplogroup tree specific to the Chinese population for forensic pedigree searches. The tree contained 139 Y-SNPs and was constructed based on NGS and pyrosequencing. Rather than applying a fixed minimum number of detected loci for exclusion, we developed a flexible likelihood formula FSindex for biostatistical support. All of the retrieved pedigrees with identical Y-SNP genotypes with crime scene evidence will go through the FSindex. Only pedigrees with FSindex greater than 1 will be treated as the potential source of the suspect pedigree. In the actual case, we narrowed down the investigation scope and successfully identified the suspect’s pedigree. This example demonstrates the power of the new approach for excluding most of the unrelated pedigrees through the high-resolution Y-SNP haplogroup analysis. Most importantly, the FSindex helps to generate positive identification of pedigrees from mismatched Y-STR haplotypes and guide further investigations.

The new approach is based on the ‘NGS+’ - combining NGS, CE and pyrosequencing together. NGS has great advantages in accurate typing and high throughput, and is the optimal choice for multi-samples and multi-Y-SNP detection. Taking NGS as the core removed the need to limit the number of detected Y-SNPs when constructing the high-resolution Y-SNP haplogroup tree and kept the marker set integrated in each single test, thus avoiding errors in haplogrouping. In this study, two samples from the Hui population unexpectedly carried extra mutations. Sample Hui58 belonging to haplogroup I-M170 carried a P203.1 mutation defining haplogroup O1a1a-P203.1. Another sample, Hui60, belonged to haplogroup N-M231 but was observed with a M117 mutation which defined haplogroup O2a2b1a1-M117. The exactly same Y-SNP genotype with the extra mutation is unlikely to occur among other pedigrees, so the extra variations inconsistent with original haplogroups can be regarded as distinctive features of their pedigrees and flow through generations steadily. CE was used not only to analyze Y-SYRs in the first step of a forensic pedigree search, but also as the mainstream technology nowadays in forensic autosomal STR typing for personal identification after the scope of the investigation has been determined. ‘NGS+’ brought the advantages of CE into full play and created a usable approach for police investigators.

The efficiency of the new approach depended directly on the resolution level of the Y-SNP haplogroup tree. Further sub-haplogrouping had to be carried out, because the 74 Y-SNPs panel could not have enough power of resolution. Human genome sequencing is now a mature technology, however, it is too extravagant to be used for evaluating evidence. Therefore, pyrosequencing was used to screen polymorphic Y-SNPs. 67 sets of primers were designed for pyrosequencing, and 53 Y-SNPs were observed to be polymorphic in 296 Chinese individuals. These Y-SNPs were selected for gradual subdivision testing. The development of haplogroup frequencies in each pyrosequencing test showed the effectiveness of each Y-SNP for differentiation at various levels. In accordance with that, Y-SNP density was adjusted over time. Pyrosequencing costs are much lower for single tests and the primers are much easier to be designed, making this method superior to NGS for adjusting marker sets. It is suitable for single Y-SNP detection in small size samples. Y-SNP sets can also be adjusted for pyrosequencing when the target population is changed. NGS+, which combines NGS, CE and pyrosequencing, can be deemed to be the most suitable approach to construct a reasonable and efficient forensic pedigree search approach. In the future, the entire final Y -SNP panel will be tested via NGS, therefore then, the forensic pedigree search approach will be based on NGS plus CE.

A likelihood formula, FSindex, was proposed for the NGS+-based forensic pedigree search approach to determine whether each retrieved pedigree needed a further investigation. Due to the non-recombining nature of the male-specific part of the Y chromosome, there is a link between Y-SNPs and Y-STRs in transmission between generations. Thus, FSindex couldn’t be simply compiled as the product of the respective likelihood formulas for these two markers (see Supplementary Note). The general correlation formula of continuous variables was applied to adjust the FSindex. P_AB_ reflects the correlation specific to the target population between Y-SNPs and Y-STRs. The estimate of P_AB_ is affected by many factors, such as sample size and the resolution level of the Y-SNP haplogroup analysis. In this study, based on the new analysis approach, we obtained the mean P_AB_ derived from four ethnicities as 0.9969 (Supplementary Figure S5). Then, we increased the sample size of one ethnicity to 517 (Supplementary Figure S6), resulting in P_AB_ = 0.9945. In the YHRD, there were over 18,000 profiles available for the P_AB_ estimate and the P_AB_ was calculated to be 0.9989, though the resolution level of the Y-SNP haplogroup tree in the YHRD was lower than the one we constructed. Therefore, it is reliable to believe that the range of P_AB_ is between 0.99 and 0.9999 for Chinese population, and the larger the sample size is or the higher the resolution level of the Y-SNP haplogroup analysis is, the closer the P_AB_ will be to 0.99. In this study, P_AB_ was set to 0.9969.

The variable X, which is equal to the average Y-STR mutation rate per generation times the maximum meiosis of the involved pedigree, reflecting the possibility that the presumed mutation must have occurred in a certain number of meiosis. The probability of mutation increased with the number of meiosis^31^. To determine the number of meiosis in each retrieved pedigree is critical, both theoretically and empirically. Though the relationship between the suspect and the representatives from the reference pedigree cannot be predicted *a prior*, we are able to obtain the maximum number of meiosis in each pedigree accordingly to the known genealogy. After this correction, the FSindex will be much more conservative. If the value is still greater than 1, it is reasonable to infer that the pedigree maybe is the suspect’s pedigree and further investigation is needed to confirm. To minimize the interference on forensic pedigree searches due to possible Y-STR mutations, it is necessary to adjust X and carry out specific estimates for each retrieved pedigree. Since the number of potential pedigrees after haplogroup screening will be very limited, adjusting the X value doesn’t complicate the calculation but enables the FSindex formula to be more general and accurate. The FSindex takes into account the Y-STR haplotype frequency and the Y-STR mutation rate, as well as the resolution level of the Y-SNP haplogroups and the haplogroup frequency in the target population. In addition, it also quantifies the correlation between the haplogroup and haplotype, rendering the formula more appropriate for forensic pedigree searches. Evaluating all the retrieved pedigrees via an FSindex avoids the subjective judgment by investigators due to Y-STR mismatches, and more reliably indicates the possibility of the involved pedigree as the suspect’s source. It provides solid biostatistical support for forensic pedigree searches to narrow down the scope of the investigation. It is worth noting that, since the FSindex dependent on the number of Y-STR mismatches in the case of the identical haplogroup, it is not appropriate for likelihood comparison between reference pedigrees.

## Methods

### Ethics statement

According to the distribution of Y chromosome variation along administrative as well as ethnic divisions in the mainland territory of the People’s Republic of China^30^, blood samples of 296 unrelated healthy male individuals including 127 Han, 47 Tibetan, 62 Uygur and 60 Hui were collected upon approval of the Ethics Committee at the Institute of Forensic Medicine, Sichuan University with informed consent. All the methods were carried out in accordance with the approved guidelines of Institute of Forensic Medicine, Sichuan University. The Ethics Committee of Sichuan University approved this study.

### DNA samples and control samples

DNA was extracted by salting out method or with the QIAamp DNA Blood Mini Kit (Qiagen, Hilden, Germany). Typical control DNA of 2800 M (Promega, USA), 9948 (Qiagen, Hilden, Germany) and 007 (Life Technologies, USA) human cell line samples were used as reference samples in every single run.

### Marker selection of NGS+

All the candidate Y-SNPs were obtained from the Y Chromosome Haplotype Reference Database (YHRD) (https://yhrd.org), International Society of Genetic Genealogy (ISOGG) (http://www.isogg.org/tree/index.html) and the 1000 Genomes Project (https://www.ncbi.nlm.nih.gov/variation/tools/1000genomes/). The Y-SNP inclusion criteria for initial genotyping on the Ion Torrent PGM^TM^ system (Life Technologies, USA) are as follows: (1) Y-SNPs of the major haplogroups in Chinese population and the marker number of each haplogroup is determined on the haplogroup frequency, (2) phylogenetically key intermediate Y-SNPs which increase integrity of the Chinese-specific tree, (3) primers could be designed for NGS and amplicons are smaller than 200 bp. Finally, 74 Y-SNPs providing the basic structure of the haplogroup tree for forensic use were screened out but two were excluded according to the latest ISOGG Y-SNP haplogroup tree version 2017.

The additional candidate Y-SNPs for further subdivision were manually selected according to the following criteria: (1) polymorphic in Chinese populations, and (2) primers could be designed for Pyrosequencing and amplicons are smaller than 150 bp. Finally, 67 additional Y-SNPs for the expansion of our forensic haplogroup tree were screened out. Details of these additional Y-SNPs are shown in Supplementary Table S2.

### Primer preparation of NGS+

AmpliSeq Designer was employed to conduct single-pool DNA Hotspot designing, and the 74 candidate Y-SNPs were submitted to Thermo Fisher AmpliSeq primer design tool (http://www.ampliseq.com) in a BED file (Thermo Fisher White Glove Approach ID: IAD97812).

Amplification and sequencing primers of the 67 additional Y-SNPs were self-designed by the PSQ Assay Design software (version 1.0, Biotage AB, Sweden). One of each pair of amplification primers was biotinylated at the 5’-end and purified by HPLC to eliminate free biotins (details in Supplementary Table S2).

### Y-SNP typing with NGS

The initial haplogroup determination of 74 Y-SNPs was conducted on the Ion Torrent PGM^TM^ system (Life Technologies, USA). Experimental steps included library preparation, emulsion PCR and sequencing.

#### Library preparation

10 ng of genomic DNA was used for library preparation using the Ion AmpliSeq^TM^ Library Kit 2.0 (Life Technologies, USA) on a ProFlex^TM^ 96-well PCR System (Thermo Fisher Scientific, USA). The library-PCR system were performed in 20 μl total volume each containing: 4 μl of 5X Ion AmpliSeq^TM^ HiFi Mix; 10 μl of 2X Ion AmpliSeq^TM^ Primer Pool; 5 μl of Nuclease-free Water; 1 μl of 10 ng/μl genomic DNA dilution. Thermal cycling consisted of: enzyme activation of 2 min at 99 °C; followed by 19 cycles of 15 sec at 99 °C, 4 min at 60 °C; finally 10 °C for hold. The PCR products were treated with 2 μl FuPa (Life Technologies, USA) to partially digest primer sequences and were continued in the thermal cycler with 10 min at 50 °C; 10 min at 55 °C; 20 min at 60 °C; and finally a 10 °C hold. Considering that multiple libraries would test on a single chip, all libraries were assigned a unique barcode using the Ion Xpress^TM^ Barcode Adapters (Life Technologies, USA) following the manufacturer recommendations. Then the ligation reaction was performed by adding 4 μl of Switch Solution (Life Technologies, USA); 2 μl of diluted barcode adapter mix prepared before addition; 2 μl of DNA Ligase (Life Technologies, USA) to each digested sample in 30 μl total volume and loaded in the thermal cycler following 30 min at 22 °C; 10 min at 72 °C; and a 10 °C hold. After purifying the unamplified library using Agencourt® AMPure® XP Reagent (Life Technologies, USA), the unamplified Ion AmpliSeq^TM^ library was eluted in 50 μl of Low TE Buffer (Life Technologies, USA). Library quantification was conducted on the 7500 Real Time PCR System (Life Technologies, USA) with Ion Library TaqMan^TM^ Quantitation kit (Life Technologies, USA) following 2 min at 95 °C and 40 cycles of 15 sec at 95 °C with the Ion Library Quantitation Kit (Life Technologies, USA). All the libraries were unified into a same concentration and the final library was established by collecting 5 μl dilutions from each dilution library.

#### Emulsion PCR

The final library was attached to Ion Sphere^TM^ Particles (ISP). Emulsion PCR (emPCR) was conducted using Ion PGM^TM^ Hi-Q OT2 Kit on the Ion One Touch^TM^ 2 System (Life Technologies, USA) following the recommended protocol. The template-positive ISPs were enriched on the Ion One Touch^TM^ ES complying with the description in the user guide.

#### Sequencing and data analysis

Finally, sequencing was performed on the Ion Torrent PGM^TM^ system (Life Technologies, USA) with the Ion PGM^TM^ Sequencing 200 kit v2 (Life Technologies) using the Ion 318^TM^ Chip V2 BC. A final volume of 30 μl was loaded onto the chip, containing sequencing primer, Control Ion Spheres^TM^ Particles and sequencing polymerase of the Ion PGM^TM^ sequencing kit (Life Technologies, USA). These runs were set in order to achieve a 500X coverage for each sample. We took Hg19 as the human reference genome. Raw sequencing data were collected as DAT files which were processed on the Torrent_Suite Server (v4.6). VariantCaller (v4.6.0.7) and CoverageAnalysis (v4.6.0.3) plugins. The BAM files were verified using IGV_2.3.59.

### Y-SNP typing by pyrosequencing

Additional 67 Y-SNPs were analyzed by pyrosequencing. Below is a step-by-step summary of the experimental steps.

#### Amplification

The PyroMark PCR Kit (QIAGEN, Germany) was used for amplification according to the manufacturer’s recommendations on a ProFlex^TM^ 96-well PCR System (Thermo Fisher Scientific, USA). Thermal cycling comprised: enzyme activation of 15 min at 95 °C; followed by 50 cycles of 30 s at 94 °C, 30 s at 62 °C and 30 s at 72 °C; final extension of 10 min at 72 °C and hold at 10 °C. Non-specific amplicons were not observed by electrophoresis in 6% polyacrylamide gels with silver nitrate staining.

#### Single strand PCR products preparation

For pyrosequencing 25 μl of the biotin-labeled PCR amplicons, 47 μl PyroMark Binding Buffer (QIAGEN, Germany) and 3 μl Streptavidin Sepharose^TM^ High Performance (GE Healthcare, Sweden) were mixed and vortexed for 10 min at room temperature. The dynabeads were adsorbed to the filter tips using a microfilter and then placed in 70% alcohol for 5 s, PyroMark Denaturation Solution (QIAGEN, Germany) for 5 s, and 10-fold diluted PyroMark Washing Buffer, 10X (QIAGEN, Germany) for 10 s using the PyroMark Q96 Vacuum Workstation (Biotage AB) for single strand PCR products preparation. After turning off the vacuum workstation, the microfilter was placed into the PSQ96 Plate Low (Biotage AB, Sweden) containing 2 μl the PSQ primer and 43 μl PyroMark Binding Buffer (QIAGEN, Germany). The plate was placed in an incubator at 80 °C for 2 min and then cooled to room temperature.

#### Sequencing and data analysis

Finally, the PCR amplicons were sequenced in a PyroMark Q96 ID system (Biotage AB, Sweden). For an obvious qualification result, the PyroMark ID 1.0 software (Biotage AB, Sweden) was utilized to arrange the sequencing dispensation order of nucleotides with the SNP mode.

### Y-STR typing with CE

DNA amplification of all the individuals was performed using the AmpFLSTR Yfiler^TM^ Kit (Thermo Fisher Scientific, USA), the AmpFLSTR Yfiler^TM^ Plus Kit (Thermo Fisher Scientific, USA) and the AGCU Y SUPP (AGCU ScienTech Incorporation, China) on a ProFlex^TM^ 96-well PCR System (Thermo Fisher Scientific, USA) according to the manufacturer’s recommendations. The PCR products were separated by CE on the ABI 3500xl Genetic Analyzer (Applied Biosystems, USA) and all haplotypes were determined with GeneMapper ID v3.2 software (Thermo Fisher Scientific). Four samples failed to produce genotypes.

### Data Availability

The authors declare that the main data supporting the findings of this study are available within the article and its Supplementary Information. Extra data are available from the corresponding author upon request.

## Electronic supplementary material

Supplementary Information

Supplementary Table S1
