# Supplementary material for: Next Generation Sequencing Plus (NGS+) with Y-chromosomal Markers for Forensic Pedigree Searches
Source: Sci Rep. 2017 Sep 12;7:11324. doi: 10.1038/s41598-017-11955-x (PMC5595879; doi:10.1038/s41598-017-11955-x)
Supplement: Supplementary file 1 — Supplementary Information [file 41598_2017_11955_MOESM1_ESM.pdf]

**Supplementary Note, Supplementary Figures S1-S6 and Supplementary Table S1- S2**

# Next Generation Sequencing Plus (NGS+) with Y-chromosomal Markers for Forensic Pedigree Searches

Xiaoqin Qian[1]*, Jiayi Hou[2]*, Zheng Wang[1]*, Yi Ye[1], Min Lang[1], Tianzhen Gao[1], Jing Liu[1], Yiping Hou[1#]

[1]Institute of Forensic Medicine, West China School of Basic Science and Forensic Medicine, Sichuan University, Chengdu 610041, China

[2]Clinical and Translational Research Institute, University of California, San Diego, La Jolla, CA 92093, USA

*These authors contributed equally to this work

[#]Corresponding author:

Yiping Hou

2nd Floor, Fa Yi Building, No.16, Section 3, Renmin Nan Road, Chengdu, China

Tel: +86−28−85501549

Fax: +86−28−85501549

Email address: profhou@yahoo.com, forensic@scu.edu.cn

**Supplementary Note**

Exact likelihood calculations of Y-SNP haplogroup were carried out following the exact approach of Kayser et al.[1] and the analysis of Y-STR mutations was carried out under a systematic reduction of the genotypic information used as Haas et al.[2] introduced：

1. Likelihood formula for Y-STR mutations

$$\frac{L(H_A:D)}{L(H_0:D)}=\frac{\binom{t}{m}\cdot(1-X)^{(t-m)}\cdot X^m}{A} \qquad (1)$$

Where t is the total number of Y-STR loci used, m is the number of mismatched Y-STR loci, and each mismatch is calculated as one repeat unit difference. X equals Y-STR average mutation rate per generation times the maximum meiosis of the involving pedigree, and the former is set as 0.005[3, 4, 5]. A denotes the Y-STR haplotype frequency of the reference pedigree in the population. The police investigators usually deem living paternal relatives within five generations to be a pedigree.

2. Likelihood formula for Y-SNP haplogroup

$$\frac{L(H_A:D)}{L(H_0:D)}=\frac{B\cdot S^n}{B^2}=\frac{S^n}{B} \qquad (2)$$

Where n is the number of Y-SNPs used, S equals one minus Y-SNP average mutation rate of 0.000000001[6], since all the retrieved pedigrees share an identical Y-SNP typing with the crime scene evidence. B denotes the Y-SNP haplogroup frequency of the population.

Based on formula (1) and (2), we proposed the FSindex likelihood formula for the forensic pedigree searches as follows:

$$\text{FSindex} = \frac{L(H_A : D)}{L(H_0 : D)} = \frac{\binom{t}{m} \cdot (1-X)^{(t-m)} \cdot X^m \cdot S^n}{A \cdot B + P_{AB}\sqrt{A(1-A) \cdot B(1-B)}}$$

Where $P_{AB}$, equal to 0.9969, obtained by averaging correlation coefficients of the four studied ethnicities.

In the forensic pedigree searches, likelihood ratios obtained by formula (1) and formula (2) less than respective thresholds were the prerequisites for FSindex use:

| | FALSE (mutation too high) | TRUE |
|---|---|---|
| Test for Y-STR | Formula (1) > Threshold A1 | Formula (1) ≤ Threshold A1 |
| Test for Y-SNP | Formula (2) > Threshold A2 | Formula (2) ≤ Threshold A2 |

We set the threshold A1 as 1, indicating that there was no potential pedigree sharing an identical Y-STR haplotype with the crime scene evidence and the number of mismatched alleles might be beyond our expectation.

**References**


1. Kayser M, Vermeulen M, Knoblauch H, Schuster H, Krawczak M, Roewer L. Relating two deep-rooted pedigrees from Central Germany by high-resolution Y-STR haplotyping. *Forensic Sci Int-Gen* **1**, 125-128 (2007).

2. Haas C*, et al.* Y-chromosomal analysis identifies the skeletal remains of Swiss national hero Jorg Jenatsch (1596-1639). *Forensic Sci Int Genet* **7**, 610-617 (2013).

3. Gusmao L, *et al.* Mutation rates at Y chromosome specific microsatellites. *Hum Mutat* **26**, 520-528 (2005).

4. Dupuy BM, Stenersen M, Egeland T, Olaisen B. Y-chromosomal microsatellite mutation rates: Differences in mutation rate between and within loci. *Hum Mutat* **23**, 117-124 (2004).

5. Kayser M, *et al.* Characteristics and frequency of germline mutations at microsatellite loci from the human Y chromosome, as revealed by direct observation in father/son pairs. *Am J Hum Genet* **66**, 1580-1588 (2000).

6. Wei W, *et al.* A calibrated human Y-chromosomal phylogeny based on resequencing. *Genome Res* **23**, 388-395 (2013).

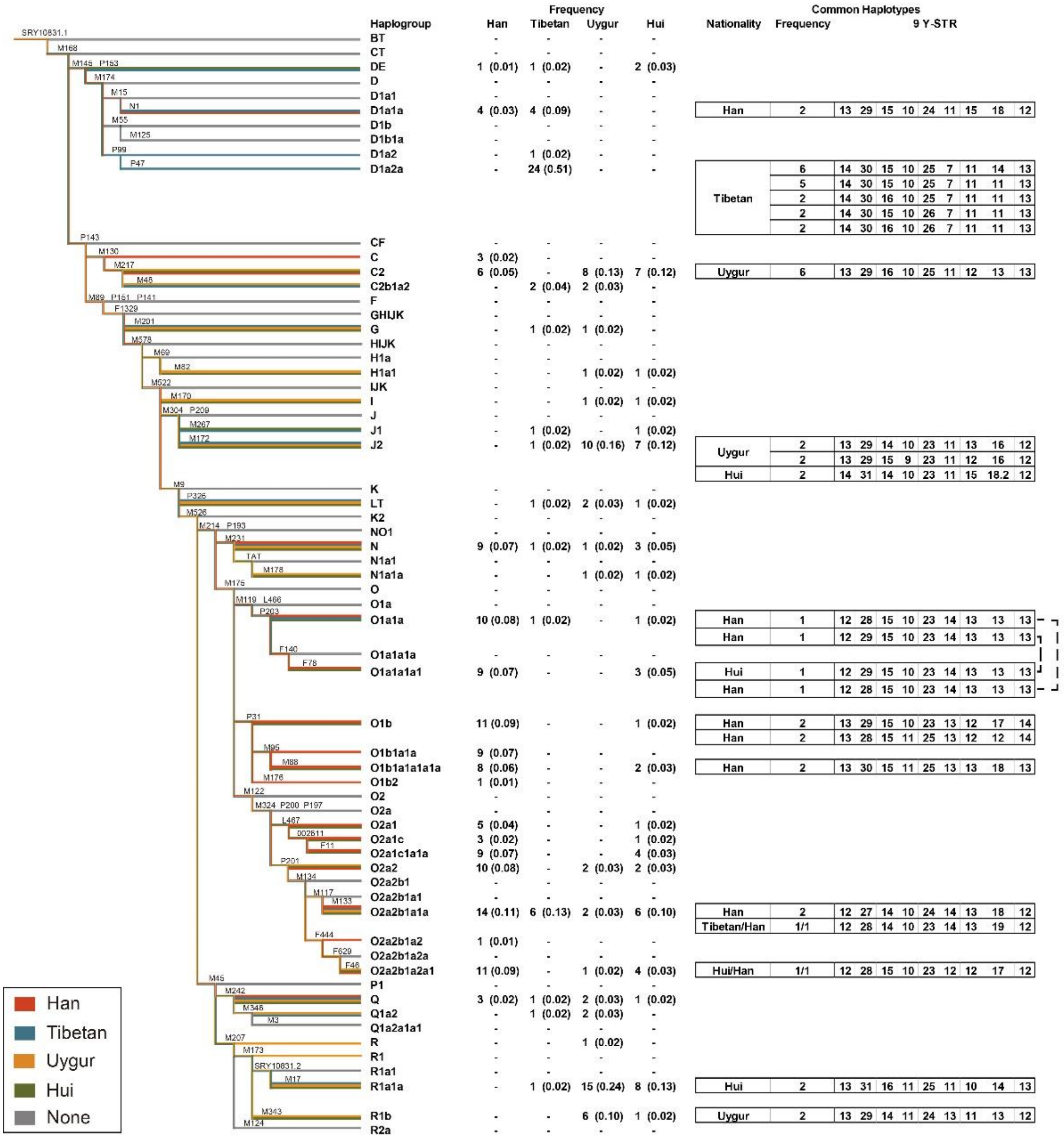


**Supplementary Figure S1. Haplotype sharing of 9 Y-STRs based on the haplogroup distribution of 296 unrelated individuals at moderate resolution.** The 72 Y-SNP markers define 63 terminal haplogroups. The corresponding haplogroups of the tested Y-SNPs and the observed frequencies of Han, Tibetan, Uygur and Hui ethnicities are shown in the middle. On the right side, Y-STR loci are arranged in the following order: DYS389I, DYS389II, DYS19, DYS391, DYS390, DYS392, DYS385, and DYS393. Only samples sharing identical haplotypes are presented with genotypes. A total of 20 groups involving 51 individuals were identical at minimal haplotypes. 3 of the 20 groups were from different ethnicities and 2 of the 20 groups were discordant at haplogroups depicted in dotted line.

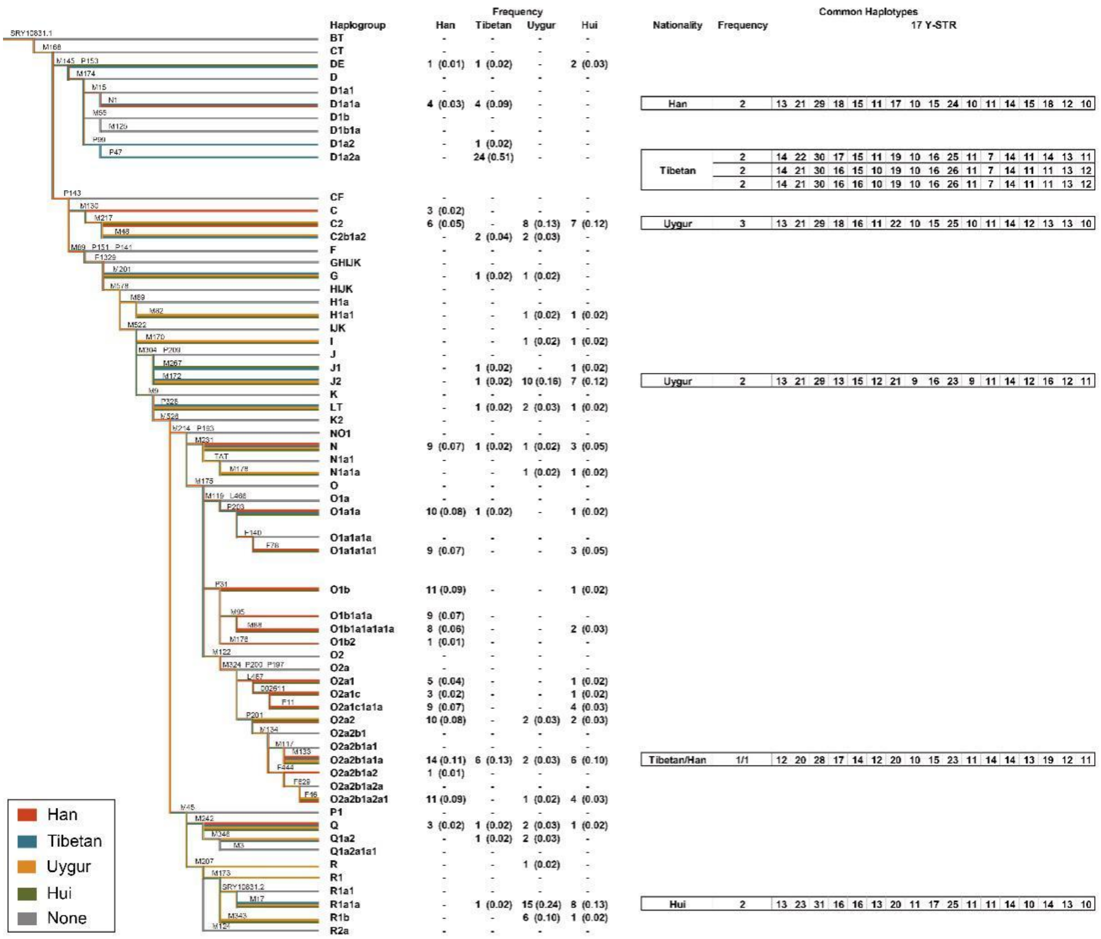


**Supplementary Figure S2. Haplotype sharing of 17 Y-STRs based on the haplogroup distribution of 296 unrelated individuals at moderate resolution.** The 72 Y-SNP markers define 63 terminal haplogroups. The corresponding haplogroups of the tested Y-SNPs and the observed frequencies of Han, Tibetan, Uygur and Hui ethnicities are shown in the middle. On the right side, Y-STRs are arranged in the following order: DYS389I, DYS635, DYS389II, DYS458, DYS19, Y_GATA_H4, DYS448, DYS391, DYS456, DYS390, DYS438, DYS392, DYS437, DYS385, DYS393, and DYS439. Only samples sharing identical haplotypes are presented with genotypes. A total of 8 groups involving 17 individuals were identical at Yfiler haplotypes. 1 of the 8 groups was from different ethnicities.

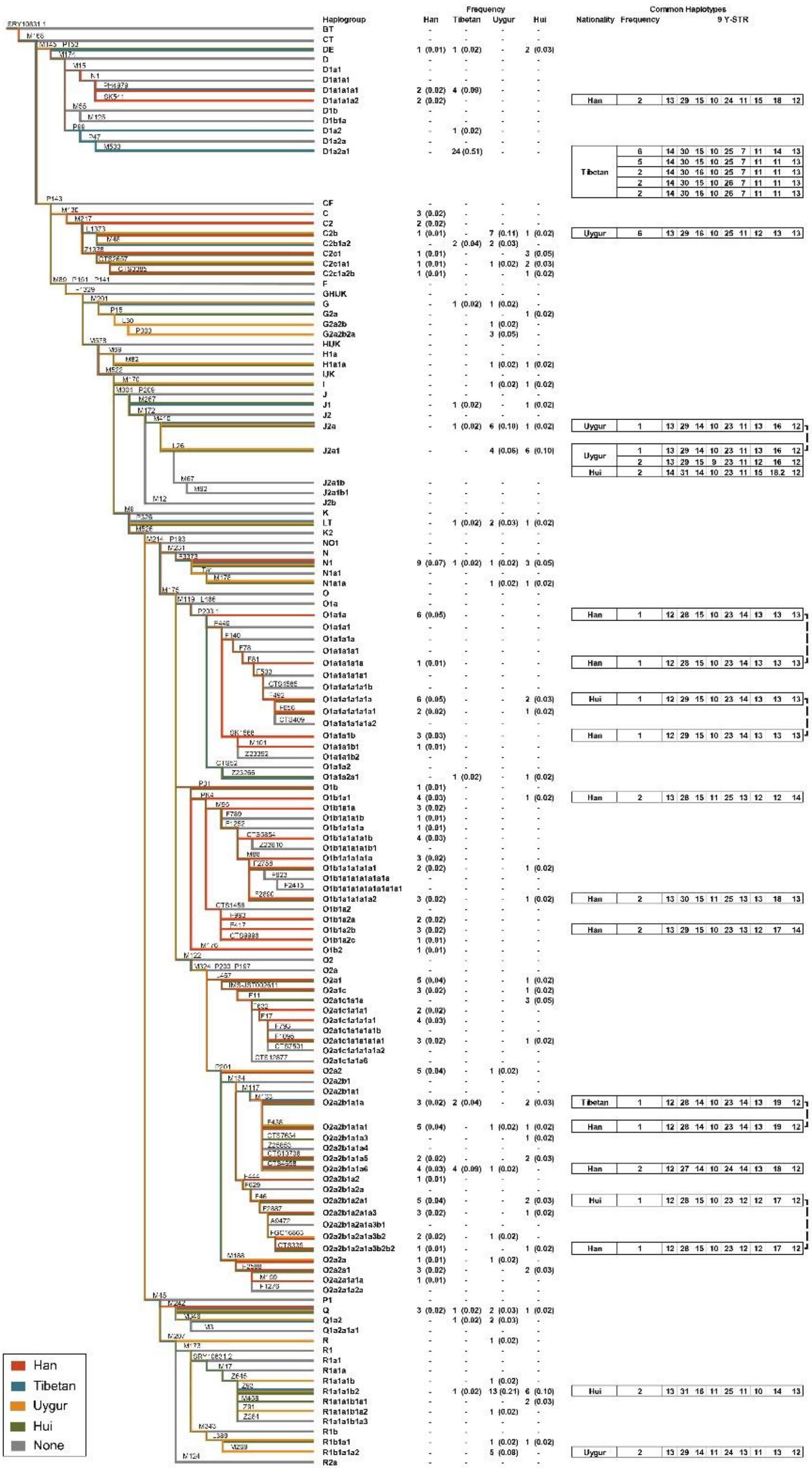


**Supplementary Figure S3. Haplotype sharing of 9 Y-STRs based on the haplogroup distribution of 296 unrelated individuals at high resolution.** The 139 Y-SNP markers define 130 terminal haplogroups. The corresponding haplogroups of the tested Y-SNPs and the observed frequencies of

Han, Tibetan, Uygur and Hui ethnicities are shown in the middle. On the right side, Y-STRs are arranged in the following order: DYS389I, DYS389II, DYS19, DYS391, DYS390, DYS392, DYS385, and DYS393. Only samples sharing identical haplotypes are presented with genotypes. A total of 20 groups involving 51 individuals were identical at minimal haplotypes. 5 of the 20 groups were discordant at haplogroups depicted in dotted line.

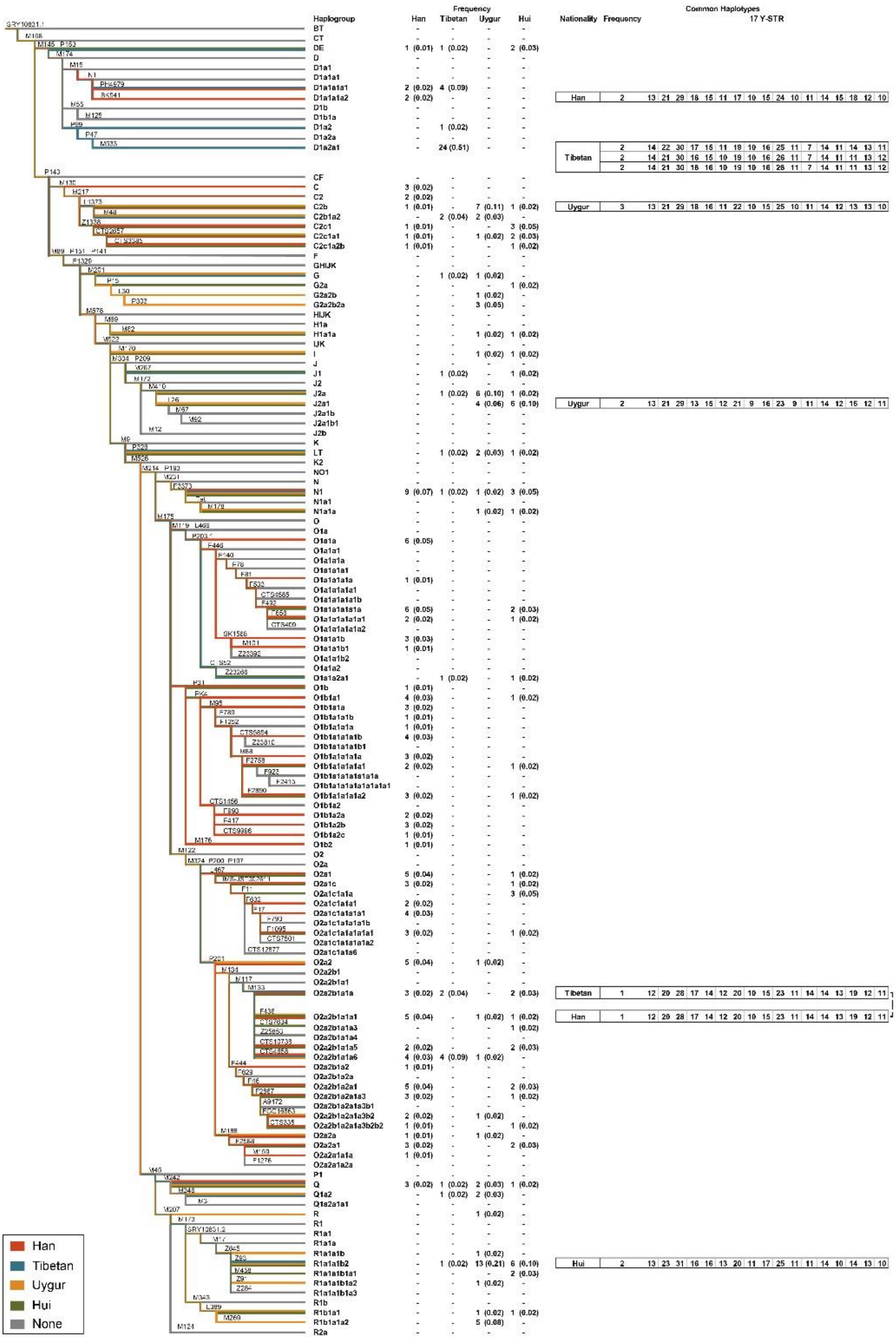


**Supplementary Figure S4. Haplotype sharing of 17 Y-STRs based on haplogroup distribution of the 296 unrelated individuals at high resolution.** The 139 Y-SNP markers define 130 terminal haplogroups. The corresponding haplogroups of the tested Y-SNPs and the observed frequencies of

Han, Tibetan, Uygur and Hui ethnicities are shown in the middle. On the right side, Y-STRs are arranged in the following order: DYS389I, DYS635, DYS389II, DYS458, DYS19, Y_GATA_H4, DYS448, DYS391, DYS456, DYS390, DYS438, DYS392, DYS437, DYS385, DYS393, and DYS439. Only samples sharing identical haplotypes are presented with genotypes. A total of 8 groups involving 17 individuals were identical at Yfiler haplotypes. 1 of the 8 groups was discordant at haplogroups depicted in dotted line.

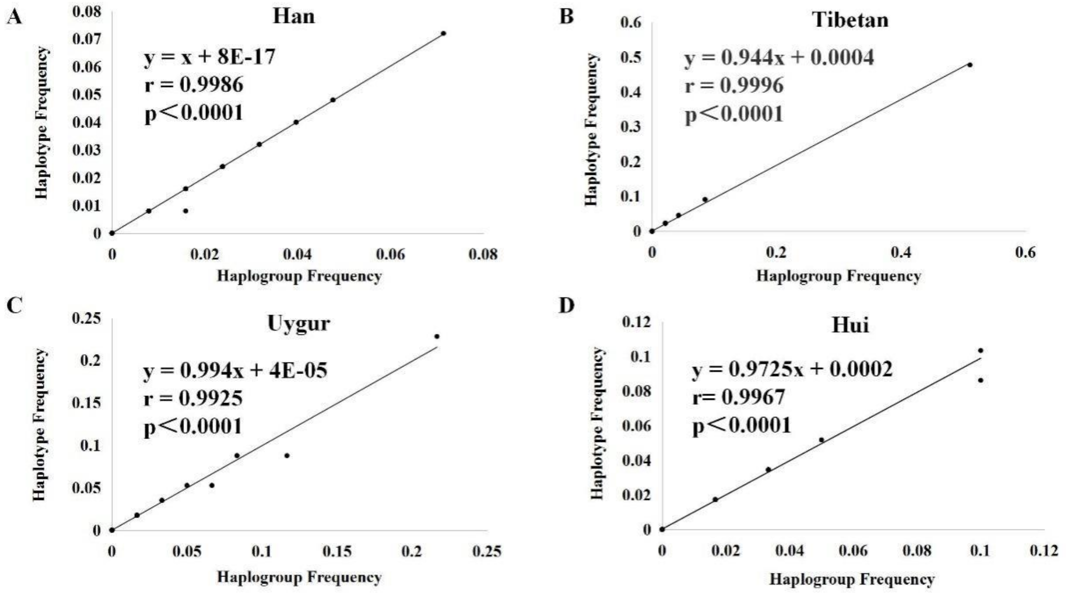


**Supplementary Figure S5. Correlation analysis of the four studied ethnicities.** **A**: A total of 124 haplotypes were observed in 126 Han individuals (1 sample was failed in genotyping), $P_{AB\text{-}Han}$=0.9986, p<0.0001; **B**: A total of 44 haplotypes were observed in 47 Tibetan individuals, $P_{AB\text{-}Tibetan}$=0.9996, p<0.0001; **C**: A total of 57 haplotypes were observed in 62 Uygur individuals (2 samples were failed in genotyping), $P_{AB\text{-}Uygur}$=0.9925, p<0.0001; **D**: A total of 58 haplotypes were observed in 60 Hui individuals (1 sample was failed in genotyping), $P_{AB\text{-}Hui}$=0.9967, p<0.0001.

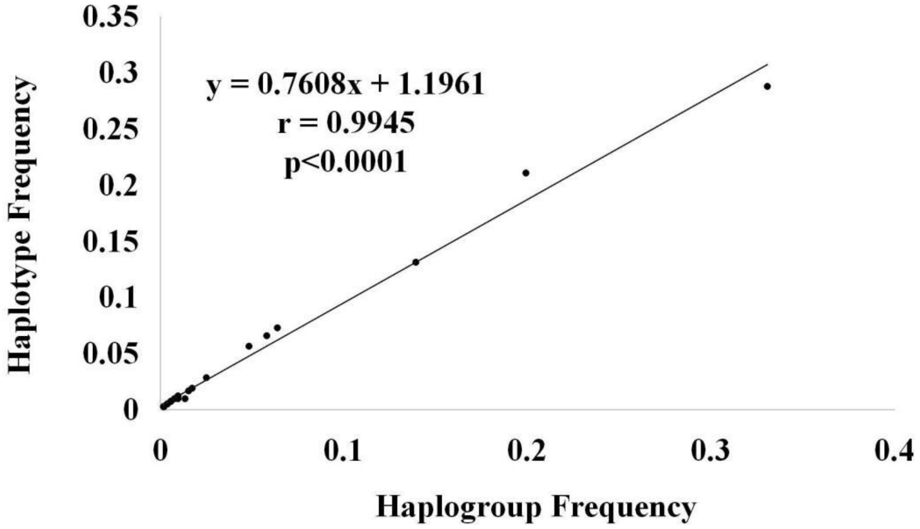


**Supplementary Figure S6. Correlation analysis of 517 profiles derived from one of the studied ethnicities.** A total of 428 haplotypes were observed in 517 unrelated males from one of the studied ethnicities. Correlation analysis was carried out and $P_{AB}$=0.9945, $p<0.0001$.

**Supplementary Table S1. Mutation count for the 41 Y-STR markers studied in 100 father-son pairs. (Mutations are in bold)**

See separate xlsx-document (Supplementary Table S1.xlsx)

**Supplementary Table S2. Primer Information of the additional 67 Y-SNPs.**

| Marker | Variation ID | Primer sequence (5′-3′) | |
|---|---|---|---|
| | | PCR Forward | AACCAGCTCACAATTTGCATTAA |
| PH4979 | rs751239817 | Biot-PCR Reverse | GTGGTTCTGCTGTTATTGCTAAAA |
| | | Seq Primer | TTAATGACAACTTAGGGC |
| | | Biot-PCR Forward | CAAGCCCCATGTACTTACCTCAA |
| SK541 | - | PCR Reverse | AGGAGGGCCAACTCATAACGT |
| | | Seq Primer | CCAACTCATAACGTGAAG |
| | | PCR Forward | AGCACAGGCAAGAGAATATGG |
| M533 | - | Biot-PCR Reverse | TGGCAGGTACTGGAGGTTGT |
| | | Seq Primer | CAGGCAAGAGAATATGG |
| | | PCR Forward | GAGTGGAGTTAAAAATGCTTTCAT |
| L1373 | rs866573363 | Biot-PCR Reverse | GTTTGCCCTTAATCATTTTCTCA |
| | | Seq Primer | AAAGCCTGCAGAGGA |
| | | PCR Forward | TGGGCATTTGTAAGAGAATGAAT |
| Z1338 | rs762747982 | Biot-PCR Reverse | TAGTGAAATTCCTGGAGAGAGCA |
| | | Seq Primer | TGATGTCTTTCTCCACCTA |
| | | PCR Forward | GGCTAGGAGGAGCTGGGAAAGTA |
| CTS2657 | rs755171620 | Biot-PCR Reverse | GATGGTAGAGTTCGGCCTCCCT |
| | | Seq Primer | GAGCTGGGAAAGTAGAAG |
| CTS3385 | rs770764944 | Biot-PCR Forward | TTTTGGAATAAAGCCACTGTCA |

| | | | |
|---|---|---|---|
| | | PCR Reverse | GGCTTCCACTTTCTCTTAGCACTC |
| | | Seq Primer | TGACAATGAAAGGAAAAA |
| | | PCR Forward | ATAGAGCCAATGCTTGAGGTTCTG |
| P15 | rs370167410 | Biot-PCR Reverse | CCAGCCCACTTGTGGACTTTATC |
| | | Seq Primer | GCTTGAGGTTCTGAATCTT |
| | | PCR Forward | TATTTGCCTGAAACTGGCTGGAAG |
| L30 | rs34134567 | Biot-PCR Reverse | GGTTGGAAATTTAGGCAAGACCAT |
| | | Seq Primer | ACTTTGATTTTTTTGGTG |
| | | PCR Forward | TAGTTCCTTTGCCATTCATGC |
| P303 | rs72625365 | Biot-PCR Reverse | CAGGCCAAGAACTAATGAAGAGA |
| | | Seq Primer | AAAGAACTTTGCTGTTGTA |
| | | PCR Forward | ATCTTTGGGAGAAACATCTTGAGT |
| M410 | rs371079691 | Biot-PCR Reverse | ATGCAGGCTGGAAGAGTAGCTAA |
| | | Seq Primer | AAACATCTTGAGTTTCTG |
| | | Biot-PCR Forward | CATCCGACTTACTATTGCTGAAGA |
| L26 | rs34459399 | PCR Reverse | GTAATTTAGCCACTGCTCTGTTTG |
| | | Seq Primer | GCCACTGCTCTGTTTG |
| | | Biot-PCR Forward | CAAGTACGTGTCCTAAAAGAAAAA |
| M67 | rs2032628 | PCR Reverse | TTTTCACTTGTTCGTGGACC |
| | | Seq Primer | CGTGGACCCCTCTATA |
| M92 | rs2032648 | PCR Forward | CGTCTTAGGATCAACATCATGTCT |

| | | | |
|---|---|---|---|
| | | Biot-PCR Reverse | GAGTCTAAATACTGTTGGAGCCTA |
| | | Seq Primer | TGGCTTGACCAAAAATA |
| | | Biot-PCR Forward | GCCACCACCCCTAGCTAATTTT |
| M12 | rs3903 | PCR Reverse | TCCAAGACTAGCCTGAGCAACATA |
| | | Seq Primer | AACATAGTGACCCCCA |
| | | Biot-PCR Forward | TTGCCTGCCATTGTCTTCTA |
| F3373 | rs34581739 | PCR Reverse | AAGACATTCCCTCAGCTTGTG |
| | | Seq Primer | TCAGCTTGTGAGGACC |
| | | Biot-PCR Forward | CTGCAAGCTTCACATTTTGTAAA |
| CTS52 | rs756720328 | PCR Reverse | GGCCAATTAAAATAACACAGAAGA |
| | | Seq Primer | TTTCTTGCTTAAGTTCCAT |
| | | PCR Forward | GACACTCAACAGTAGCCTGTGAAA |
| Z23266 | rs763653967 | Biot-PCR Reverse | GAGGGTGGTTCCTATGGTCTTAGA |
| | | Seq Primer | AGGGAGGCTGCACGC |
| | | Biot-PCR Forward | GGAAGGCCTCCGTTTCAC |
| F789 | rs375155527 | PCR Reverse | TGATACAACCCCAGTGAGAAGC |
| | | Seq Primer | ATGCATCTTTCAGTTGTC |
| | | Biot-PCR Forward | AGGTGCATATGGTGCAGACTCCT |
| F1252 | rs778762075 | PCR Reverse | TTTCAGGCTGGTTCAAGTCTGATG |
| | | Seq Primer | GACCACGGGACCATC |
| CTS5854 | rs755156680 | PCR Forward | GTGGAACATGCCTGTAATCCC |

| | | | |
|---|---|---|---|
| | | Biot-PCR Reverse | CCCAGATTCAAGTGATTCCTATG |
| | | Seq Primer | CCAGCTACTGGGGAG |
| | | Biot-PCR Forward | TCAAACTCCTGACCTCAGGTAA |
| Z23810 | rs746242336 | PCR Reverse | AGCACTTTGGGAGGCCAAG |
| | | Seq Primer | GGAGGCCAAGGCGGG |
| | | PCR Forward | AGAGAGGAGCTCATTACATGACA |
| F2758 | - | Biot-PCR Reverse | GAGGTTGTAAGGCATCACCTG |
| | | Seq Primer | GCTCATTACATGACAGATTA |
| | | Biot-PCR Forward | TCTACACTCACAGAGCAACACC |
| F923 | rs774805385 | PCR Reverse | AAAGACCGATGTTAGAATATGAA |
| | | Seq Primer | TATTAACATAGAAGTATAAA |
| | | PCR Forward | CTTTGCAATGGGTTAAATGATTC |
| F2415 | rs767441033 | Biot-PCR Reverse | GAACCCTGAACTAAAGGGAATAA |
| | | Seq Primer | TGGGTTAAATGATTCCT |
| | | Biot-PCR Forward | GACCTTGAGAAGCACAAATCAT |
| F2890 | rs745533241 | PCR Reverse | TTTTTCCAGGTTACTGGTAAGATA |
| | | Seq Primer | TTACAGGTACTTTTGTAGAC |
| | | Biot-PCR Forward | GGGTGGAAAGGGGACAGAATT |
| F632 | rs770932305 | PCR Reverse | TGCATGTGACATCCTGAACCTACT |
| | | Seq Primer | GGATTCTAAAATGTTTCCAG |
| F17 | rs774129195 | Biot-PCR Forward | GTGTTCAAATGCTAGGTGCTCTAA |

| | | | |
|---|---|---|---|
| | | PCR Reverse | CCTTGGCGTCAGTTTCATCA |
| | | Seq Primer | GCAGCCTTTGTAGGCTC |
| | | PCR Forward | GCTCTTCCTCCTCCTTCAGTTG |
| F793 | rs775936852 | Biot-PCR Reverse | GCTCTGCCTCAGTCATCTTTATAC |
| | | Seq Primer | GACATATGGACTACCTTCTG |
| | | Biot-PCR Forward | TGAATTATGTCTTGCCTGTCAAA |
| F1095 | rs54159999 | PCR Reverse | TTTTCCCATAATAGTAATGCCACA |
| | | Seq Primer | ACAAGAATTAGAAAGATACA |
| | | Biot-PCR Forward | GCACTGCCTAGTGGAGCTGTA |
| CTS7501 | rs756771282 | PCR Reverse | CTTGGGTCTGGAGGATCGT |
| | | Seq Primer | GGAGGATCGTGGCCG |
| | | Biot-PCR Forward | CTGAGATGGCCAGATAGATGCAA |
| CTS12877 | rs767939231 | PCR Reverse | AAGCTGTGGCCACCTCTATCTCC |
| | | Seq Primer | GGCCACCTCTATCTCC |
| | | Biot-PCR Forward | CAGCAAAAGCCACAGAGAAA |
| F438 | rs750131643 | PCR Reverse | CTGTAGAAAACTTTAAATCCTCCC |
| | | Seq Primer | AAAACTTTAAATCCTCCC |
| | | Biot-PCR Forward | CCTGGGCTCAAGTGATTTTC |
| CTS7634 | rs769008171 | PCR Reverse | AGCTAAAAATGGTGGCACATG |
| | | Seq Primer | TGGTGGCACATGCCT |
| Z25853 | rs747718394 | PCR Forward | CATTGCCAAGTCAATCCTAAGC |

| | | | |
|---|---|---|---|
| | | Biot-PCR Reverse | CATGCTGTTTTGGTTACTGTAGCC |
| | | Seq Primer | CTGAAAGCATCACGC |
| | | PCR Forward | ACCATAATCAAATGGAATTTGTCT |
| CTS10738 | rs542226385 | Biot-PCR Reverse | ATGGCTTAACCGAATGTCTCA |
| | | Seq Primer | GTCTTGAAATAATTTGACCT |
| | | Biot-PCR Forward | GGGCCAGTATCTTCAACTGAAA |
| CTS4658 | rs770165926 | PCR Reverse | TGTTTGCCCCATCAGTCC |
| | | Seq Primer | TTGCCCCATCAGTCC |
| | | PCR Forward | AGGTGGATTGCTGGATCATA |
| F2887 | rs764768067 | Biot-PCR Reverse | CAGAGAGCTACCTCCAAACACTAA |
| | | Seq Primer | TGGATTGCTGGATCATA |
| | | PCR Forward | CCCCCAACCCCAAACTCT |
| A9472 | rs762902463 | Biot-PCR Reverse | GTGGCTAAGCTATGCTCAACAG |
| | | Seq Primer | AACCCCAAACTCTCAC |
| | | Biot-PCR Forward | CAAGGCAAAAGAGTTACATCACC |
| FGC16863 | rs760764669 | PCR Reverse | GGGCCTATCAACTAGTCAATGG |
| | | Seq Primer | TTGGCCTCATGCACA |
| | | Biot-PCR Forward | TATGGACAAACTATGAGGCCAAAC |
| CTS335 | rs763473282 | PCR Reverse | TTGCAAAGCCAATAAAAGCCC |
| | | Seq Primer | CCAATAAAAGCCCCT |
| M188 | rs2032605 | PCR Forward | GAGCAGGCTATTCCAGTCTGTA |

| | | | |
|---|---|---|---|
| | | Biot-PCR Reverse | TCACACAGCCAATAGCTAATGC |
| | | Seq Primer | TCCAGTCTGTAGTTCACC |
| | | PCR Forward | AGCATGATTTGAGAGCATAAACTG |
| F2588 | rs376683036 | Biot-PCR Reverse | TTCTGGGATTACAGGCACAAAC |
| | | Seq Primer | TAAACTGTAAAGATCAGAAG |
| | | PCR Forward | TTTTATTATTGATGCAAGCCCTAA |
| M159 | rs72622300 | Biot-PCR Reverse | TCTGTGTTCCTTGCTCCATTAAA |
| | | Seq Primer | TGATGCAAGCCCTAA |
| | | PCR Forward | GCTTTCAATCACCACAGATG |
| F1276 | rs374901395 | Biot-PCR Reverse | GCAGAAGAGATGGACATAGTGTT |
| | | Seq Primer | AATCACCACAGATGGC |
| | | PCR Forward | ATGGGCAATATCCCTGTATAAGAA |
| Z645 | rs111731595 | Biot-PCR Reverse | CCTGTAGTGCTTTGACTCATCTCT |
| | | Seq Primer | CCTGTATAAGAACCCCAGT |
| | | Biot-PCR Forward | TAATTCACCAAACTCCCCAAATG |
| Z93 | rs566323605 | PCR Reverse | CAGGGATTCAGGAAAAGACAATTA |
| | | Seq Primer | ATTATTATTAAGGGTTTGGA |
| | | PCR Forward | TACAGCTCAGGTGCCAGGGAATA |
| M458 | rs375323198 | Biot-PCR Reverse | GAAAGACATTCCTCCTGGCTCTC |
| | | Seq Primer | AGGGAATAAATTCCATTTGA |
| Z91 | rs111908460 | PCR Forward | TACTTGAGAGGCTGAGTCAGGA |

| | | | |
|---|---|---|---|
| | | Biot-PCR Reverse | AGGCTCGAGTGCAGTGATG |
| | | Seq Primer | GGCTGAGTCAGGAGAA |
| | | Biot-PCR Forward | TGCATATTCAGCAGACAGTACAA |
| Z284 | rs767265794 | PCR Reverse | CCATTACAAAAGCCTTAATGACAC |
| | | Seq Primer | CCTACAGAATATTCGCTAGA |
| | | Biot-PCR Forward | GACCAACAGAAACAAGGACAAGTA |
| L389 | rs1358368 | PCR Reverse | CTGCCCCATCCATATACTGAGT |
| | | Seq Primer | GAACATCCATCACTTTCA |
| | | Biot-PCR Forward | AGAAGGGGAATGATCAGGGTTT |
| M269 | rs9786153 | PCR Reverse | GGCCACTATACTTCTTTTGTGTGC |
| | | Seq Primer | ATTGTTTTCAATTTACCAG |
| | | PCR Forward | GGTATCTCGCTCAGGGATTATAAA |
| F446 | rs72611639 | Biot-PCR Reverse | TAGTGCCTCCACCTCTCAGATT |
| | | Seq Primer | TGAGTATTGGCTCCTTT |
| | | PCR Forward | CAGTGAGTTCCCTTGAAATATGAT |
| SK1568 | rs746981903 | Biot-PCR Reverse | TTGGAAAACCTCTGGAGTTAGAA |
| | | Seq Primer | CCAAGTTTTGAAGTCATTAT |
| | | PCR Forward | CCCAGTTCCTGCAAAGTTAGGAGA |
| M101 | rs796569383 | Biot-PCR Reverse | GGCAATCGGAAGCCTCAATCTAT |
| | | Seq Primer | GGAGATTTACTGAATCAGTG |
| Z23392 | rs760637334 | Biot-PCR Forward | TTGAAGCTTAACAAATTTCATTC |

| | | | |
|---|---|---|---|
| | | PCR Reverse | ACAGTATAAAGCATAGGGAACAG |
| | | Seq Primer | ATAGGGAACAGTATGAAGG |
| | | PCR Forward | TTCCAAAATCGGCAATGGT |
| PK4 | rs367562925 | Biot-PCR Reverse | GGGTGTCCATAGTAACCTGTTGA |
| | | Seq Primer | GGCAATGGTTTTTGA |
| | | Biot-PCR Forward | ATTATAGGCATGAGGCACCACACA |
| CTS1456 | rs768521551 | PCR Reverse | CGGCCATGTCATTTCCTTTTTATT |
| | | Seq Primer | ATTAACATGATACAAAATGA |
| | | PCR Forward | TAAACCTTACATGCCCATTTCTTC |
| F993 | rs754430192 | Biot-PCR Reverse | CCTTTTCTGGGACAATCAGTCT |
| | | Seq Primer | ATTGATGGGACCCCT |
| | | PCR Forward | GGCAAGCTGGACAAAACTATAATG |
| F417 | rs200849799 | Biot-PCR Reverse | CAGCTGTTTCGGTTTCTAGAATAA |
| | | Seq Primer | AATTTAAGTCTTAGCCCTTC |
| | | Biot-PCR Forward | ACTGTGCTGTACATTCCACACTC |
| CTS9996 | rs774793026 | PCR Reverse | AGAAACAGTATGTCAGGCACATG |
| | | Seq Primer | CACATGGAAGGAACAGA |
| | | Biot-PCR Forward | TGCCTGTATTTTGAATTATGACCA |
| F81 | rs72618714 | PCR Reverse | CGCCATTGTTTTCATATGCACT |
| | | Seq Primer | TTAAAGCAGTTTTTCTCTGT |
| F533 | rs72611681 | Biot-PCR Forward | ATGCTGGTGGAACCCAGTGT |

| | | | |
|---|---|---|---|
| | | PCR Reverse | CGGATGCATATGTGTGGTTAGA |
| | | Seq Primer | TGTGGTTAGAAAGGACAT |
| | | PCR Forward | ACCATAGTGGAATATGCCCATT |
| CTS4585 | rs758004261 | Biot-PCR Reverse | CTTACTCATATTTCCCCTTTTTTG |
| | | Seq Primer | CCACAATTAAAAGTATACTC |
| | | Biot-PCR Forward | TTCTCCCAAGTTTGCTATCCC |
| F492 | rs72611661 | PCR Reverse | GCAAAGTTGCACGGAATTTC |
| | | Seq Primer | GGAGTACTGAGAGCAGAAT |
| | | Biot-PCR Forward | GGATGAACGGAGGCAGTGA |
| F656 | rs72615056 | PCR Reverse | GCGGGTGTCTGTGGAAGAT |
| | | Seq Primer | GTGTCTGTGGAAGATGG |
| | | PCR Forward | GGGGACTTTTTGTGGTCATTGAT |
| CTS409 | rs764765117 | Biot-PCR Reverse | GAACCCCCAGCAAATCACA |
| | | Seq Primer | TTTCAATGGTTTGATCC |
